# Two dimensional twin T-graphene: monolayer for visible-light photocatalytic water splitting and bulk for anode material of magnesium batteries

**DOI:** 10.1039/d2ra06121j

**Published:** 2022-10-24

**Authors:** Jiahe Lin, Xiaowei Chen, Bofeng Zhang, Chunrong Tan, Qiubao Lin, Xiulin Wang

**Affiliations:** School of Science, University Xiamen 361021 China linjiahe@jmu.edu.cn lqb@jmu.edu.cn; Semiconductor Industry and Technology Research Institute, Jimei University Xiamen 361021 China; Department of Chemistry, College of Chemistry and Chemical Engineering, Xiamen University Xiamen 361005 China

## Abstract

Two-dimensional (2D) carbon allotropes with all-sp^2^-hybridization have demonstrated great potential in nano-photoelectric devices, but the application of semiconductor photocatalysts for water splitting and anodes in magnesium batteries remains unoptimistic. Motivated by this, we theoretically study a highly stable all-sp^2^-hybridized 2D carbon allotrope twin T-graphene (TTG) *via* first-principles simulations. And through the calculations of the HSE06 functional, we find that TTG has a wide bandgap (2.70 eV) and suitable band edge positions satisfying the criteria of photocatalysts for overall water splitting. Additionally, TTG exhibits excellent photocatalytic properties for overall water splitting reflecting a high STH efficiency (12.34%), strong absorption coefficient in the visible-light region and the carrier mobility being high for electrons but low for holes. By investigating the strain effects, we get that, with biaxial compressive strain, the ability of overall photocatalytic water splitting can be effectively improved including STH up to ∼30%. Moreover, the bulk TTG also exhibits great potential as an anode material of magnesium batteries with a theoretical capacity of 556 mA h g^−1^, average voltage of 0.74 V and diffusion energy barrier of ∼0.96 eV. Our results would broaden the application of all-sp^2^-hybridized 2D carbon allotropes in the semiconductor photocatalytic and magnesium batteries field.

## Abstract 2

Twin T-graphene has great potential in overall photocatalytic water splitting and as the anode material of magnesium batteries.

## Graphical abstract

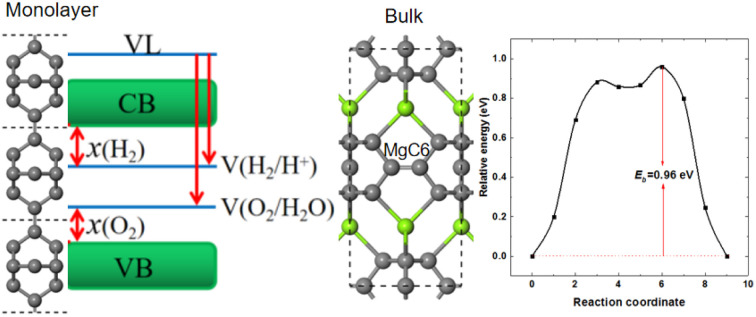

## 1. Introduction

Since the great success of monolayer graphene,^1,2^ 2D carbon allotropes have been one of the hot research topics in both the theoretical and experimental field due to their diverse layer structures, unique properties and great potential applications in the nano-device field.^3–13^ Among the discovered 2D carbon allotropes, a considerable amount of systems are planar or nearly planar with their carbon atoms bonding with each other by all-sp^2^-hybridized chemical bonds or sp + sp^2^-hybridized chemical bonds. And because the π electrons in these types of 2D carbon allotropes can flow across their surface freely but bond with difficulty with Mg atoms, almost All-sp^2^-hybridized and sp+sp^2^-hybridized 2D carbon allotropes have poor performance in the applications of semiconductor photocatalysts and magnesium batteries. For instance, graphene, T-graphene, phagraphene and α-graphyne are semi-metallic owning Dirac cones in their band structures,^14–21^ and both graphdiyne and THD-graphene are narrow-bandgap semiconductors with ∼0.48 eV intrinsic direct bandgaps,^22,23^ which can not satisfy band structure conditions for photocatalysts used in water splitting. In addition, Mg intercalation into monolayer graphene even its bulk structure are very difficult, which is also a frequent phenomenon for most All-sp^2^-hybridized and sp + sp^2^-hybridized 2D carbon allotropes.^24–26^

Recently, two types of the *P*6/*mmm*-phase All-sp^2^-hybridized 2D carbon allotropes, named twin graphene and hP-C18, respectively, have been theoretically proposed.^27,28^ The twin graphene can be viewed as AA-stacked carbon hexagons being connected together by horizontal carbon dimers, while the hP-C18 carbon can be viewed as AA-stacked carbon hexagons being connected together by vertical carbon dimers. Interestingly, unlike most other metallic or narrow-bandgap the All-sp^2^-hybridized and sp + sp^2^-hybridized 2D carbon allotropes, the twin graphene is a direct-bandgap semiconductor with a 0.981 eV bandgap, and the hP-C18 is a wide-bandgap semiconductor with a 2.93 bandgap (>2.0 eV). The significant semiconductivity of these two systems is mainly due to the carbon dimers being not coplanar with the AA-stacked carbon hexagons, which makes π electrons hard to flow across their surface unless they overcome the spatial barrier. However, the band structures of twin graphene and hP-C18 make them unsuitable for photocatalytic water splitting. Besides, twin graphene and hP-C18 also can not stably absorb Mg atoms on their surfaces. Interestingly, based on the principle of structural design, twin T-graphene (TTG) has also been proposed, which exhibited a underestimated bandgap 1.79 eV by GGA + PBE functional.^29^ Moreover, with the unsufficient evidences, its discoverers assumed that it was a potential photocatalyst for water splitting, which needed further study to clarify. Besides, the abilities of TTG in magnesium batteries also remain to be explored. Therefore, it is necessary to further study the performance of TTG on photocatalytic water splitting and magnesium batteries.

**Fig. 1:**
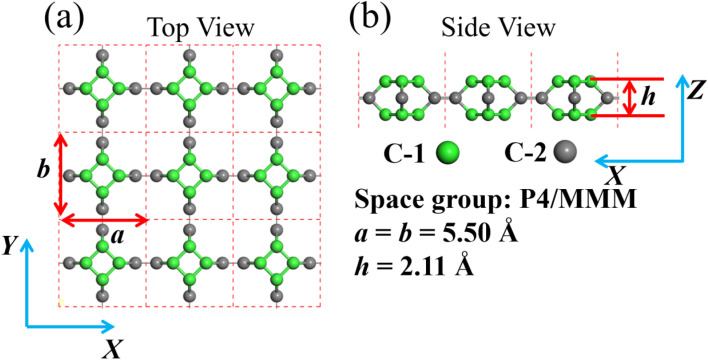
(a) and (b) are the structure model of TTG. The illustration for the lattice constants, layer height and space group of TTG is shown at the bottom right.

In this paper, we theoretically study TTG (shown in Fig. 1) based on first principles density functional theory. Through the calculations of cohesive energy, phonon spectrum and *ab inito* MD simulation, we confirm that TTG own high stability. The band structure, work function and optical absorption spectrum of TTG are studied by using HSE06 functional, which is well known that can get more accurate band structures than that by GGA + PBE functional for semiconductors. Our results indicates that TTG is an indirect wide-bandgap semiconductor and satisfies the band structure conditions of photocatalysts for visible-light overall water splitting. In addition, we also study the mechanical biaxial strain response of TTG. The strain response of electronic, optical and photocatalytic properties shows that biaxial strain can effectively tune the performance of TTG in the application on photoelectric devices and photocatalysts. Finally, we study the ability of TTG and its bulk structure for absorbing Mg atoms and relative properties as the anode of magnesium batteries. Then it reveals that bulk TTG structure has potential on the anode materials of magnesium batteries.

## 2. Computational details

All our calculations were performed by using first principle density functional theory (DFT). The CASTEP code was adopted to calculate the phonon spectrum, band structures, work function and optical absorption spectrum, while norm-conserving pseudopotential and planewave cutoff energy of 750 eV were employed.^30^ For the geometry optimization and phonon spectrum, the GGA + PBE functional (the GGA is the generalized gradient approximation, and the PBE is Perdew–Burke–Ernzerhof) was used.^31^ Our models were fully relaxed until reaching that the forces in the systems were lower than 0.01 eV Å^−1^ and the energy convergence tolerances were smaller than 1 × 10^−6^ eV per atom. 30 Å vacuum layer for monolayer TTG were adopted. The *k*-point sampling for the Brillouin zone by using Monkhorst–Pack scheme was set as 4 × 4 × 1.^32^ The phonon spectrum was carried out by the linear response method.^33^ The electronic band structures and optical absorption spectrum were got through the GGA + PBE and HSE06 (the HSE is Heyd–Scuseria–Ernzerhof) functional.^34^ Although the initial designers of TTG have performed ab inito MD simulation (AIMD) for TTG at 300 K by using a primitive cell, it is not so accurate to reveal the thermal stability of TTG. To order to further carry out its thermal stability at 300 K, we also performed AIMD calculation for TTG at 300 K with a 4 × 4 × 1 supercell, which contains 192 atoms. The AIMD simulation is performed by the Vienna ab initio simulation package (VASP) code.^35^ The simulating time is 4 ps, which is executed into 4000 steps. The 1 × 1 × 1 *k*-point setting was adopt and energy convergence tolerance was lower than 1 × 10^−4^ eV per atom. Finally, the Mg diffusion barriers were calculated by the climbing image nudged elastic band (CI-NEB) method.^36^

## 3. Results and discussion

### 3.1. Structural stability of TTG

After the full geometry optimization, the structure model of monolayer TTG is exhibited in Fig. 1(a) and (b), and the lattice constants are marked in Fig. 1(a) and (b). As shown in Fig. 1, there are two types of inequivalent carbon atoms in the unit cell, denoted as C-1 (green) and C-2 (gray), respectively. In the TTG, each carbon atom connects with two C-1 atoms and one C-2 atom to form a monolayer all-All-sp^2^-hybridized carbon allotrope. In order to confirm the stability of TTG, the cohesive energy (*E*_coh_), phonon spectrum and AIMD simulation at 300 K have been carried out. Firstly, for calculating its *E*_coh_, we adopt the following standard expression:1
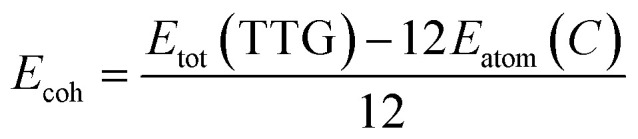
where *E*_tot_ is the total energy of fully optimized TTG, *E*_atom_(*C*) is the energy of a free carbon atom. Our calculation indicates that the *E*_coh_ of TTG is −8.01 eV per atom and lower than that of twin graphene. Therefore, we are of the opinion that this *E*_coh_ can be considered a hard evidence for proving TTG being stable in energy.

**Fig. 2:**
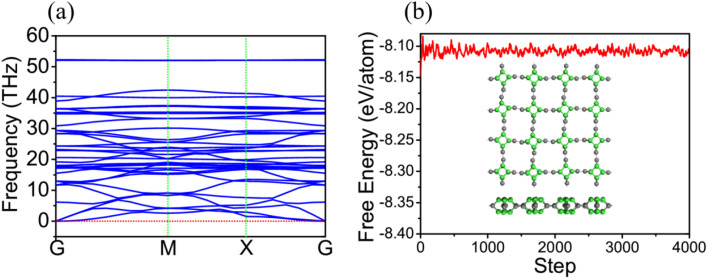
(a) is the phonon spectrum of TTG. (b) is the time evolution curve of free energy for TTG simulated by *ab inito* MD at 300 K, and the insets in (b) is the structure of TTG at 300 K.

Additionally, we have obtained the phonon spectrum of TTG shown in Fig. 2(a). Fortunately, we get that there is no imaginary frequency through the whole Brillouin zone with its minimum of the acoustic branch being linear around the Gamma point, which demonstrates that TTG is dynamically stable. Moreover, we also have performed AIMD simulation at 300 K to further study the thermal stability of TTG at room temperature. The results are shown in Fig. 2(b). We get that the time evolution curve of free energy for TTG fluctuates around the equilibrium position, and its structure, shown in the inset of Fig. 2(b), still shows no significant structural differences as compared with its initial structure. Thus, it indicates that TTG is thermally stable at room temperature.

### 3.2. Electronic properties of TTG

**Fig. 3:**
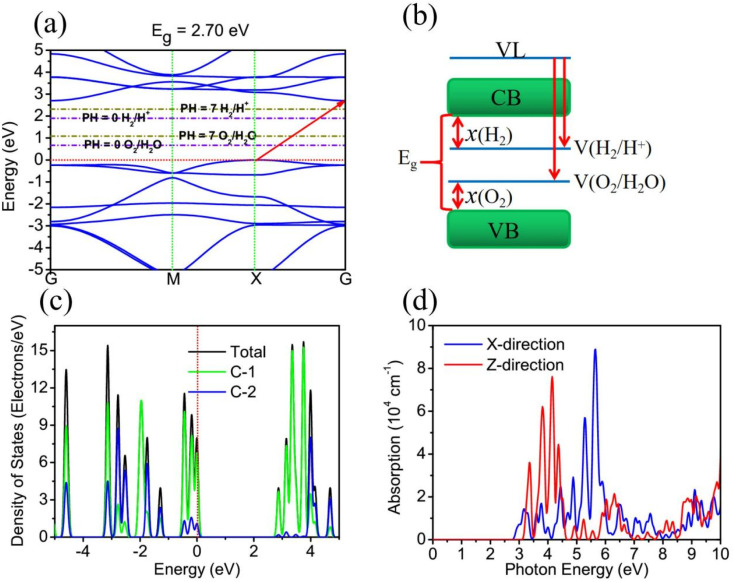
(a) is the band structure of TTG, which is compared with the redox potentials in water splitting at pH = 0 and pH = 7 shown as pink dashed lines and wathet blue dashed lines, respectively. (b) The energy level diagram for photocatalytic water splitting. (c) The TDOS and PDOS of TTG. (d) The optical absorption spectrum of TTG with the incident light polarized in *X*-direction and *Z*-direction.

To study the electronic structure properties of TTG, we have carried out its band structure calculated by HSE06 functional, as shown in Fig. 3(a) and (b), respectively. The Fig. 3(a) shows that TTG is indirect-wide-bandgap (*E*_g_ = 2.70 eV) semiconductor with its valence band maximum (VBM) lying at *X* point and conduction band minimum (CBM). This decent bandgap means that TTG have potential applications on high frequency and high power electronic devices, UV-LEDs and even photocatalysts for water splitting. However, for a 2D semiconductor that can be used for overall water splitting, its CBM energy levels relative to the vacuum level must be lower than the oxidation potential [*V*(O_2_/H_2_O)] for H_2_O to O_2_ and its VBM energy levels relative to the vacuum level must be higher than the reduction potential [*V*(H_2_/H^+^)] for H^+^ to H_2_ (the schematic diagram is shown in Fig. 3(b)). And the redox potential of water splitting depended on the pH values can be calculated by *V*(O_2_/H_2_O) = −5.67 eV + pH × 0.059 eV and *V*(H_2_/H^+^) = −4.44 eV + pH × 0.059 eV. Therefore, the *V*(H_2_/H^+^) and *V*(O_2_/H_2_O) are −4.44 and −5.67 eV at pH = 0, respectively, and the *V*(H_2_/H^+^) and *V*(O_2_/H_2_O) are −4.027 and −5.257 eV at pH = 7, respectively. Unfortunately, this criteria of photocatalysts for water splitting is always hard to satisfy for most monolayer materials, such as black phosphorene, monolayer SnSe and monolayer C_3_N. Encouragingly, comparing the CBM and VBM of TTG with the redox potentials in water splitting at pH = 0 (pink dashed lines) and pH = 7 (wathet blue dashed lines) shown in Fig. 3(a), we obtain that the band edge positions of TTG satisfy the basic band structure conditions of photocatalytic water splitting at pH = 0 and pH = 7. This reveals that TTG can be regarded as a promising candidate 2D semiconductor for overall photocatalytic water splitting.

For studying the different contribution of two types carbon atoms for the electronic occupancy states, the calculations of the total density of states (TDOS) and partial density of states (PDOS) for TTG have been performed, and the results are shown in Fig. 3(c). Learning from the TDOS of TTG, we get that the occupancy state peaks near the VBM and CBM are considerably high and the energy difference between these two peaks is ∼2.80 eV, which means that TTG would have strong optical absorption at violet-light region caused by avoiding carrier blocking effect. The PDOS of TTG indicates that both the occupied state near the VBM and CBM are mainly contributed by C-1 atoms, while the contributions from C-2 atoms are very low. Additionally, it is clear that both the occupied state of the VBM and CBM are contributed by surface C-1 atoms, which means that photogenerated charge can easier involve in photocatalytic reaction. Based on the analysis of PDOS, we find that the active π electrons have been limited in C-1 atoms and further make TTG being semiconductive.

### 3.3. Photocatalytic behaviour of TTG for overall water splitting

For an excellent photocatalyst, the ability of harvesting sunlight, particularly visible and ultraviolet light, to give rise to electron–hole pairs is crucial to water splitting.^37–40^ To determine the optical absorption coefficient of the TTG, we have calculated its optical absorption spectrums that are polarized in *X*-direction and *Z*-direction of TTG, as shown in Fig. 3(d). The frequency-dependent dielectric function that is obtained and expresses as *ε*(*ω*) = *ε*_1_(*ω*) + i*ε*_2_(*ω*), which can be used to carry out the absorption coefficient as follow:2
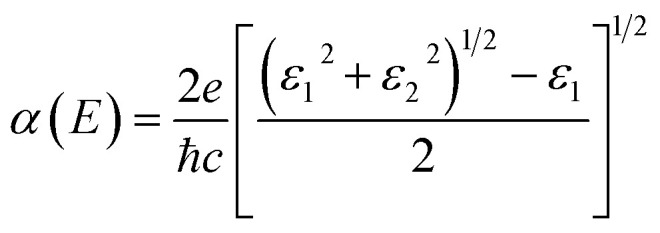


The Fig. 3(d) shows that, when the incident light is polarized in *X*-direction, TTG has strong optical absorption at the range of photon energy about 2.80–4.00 eV, and when the incident light is polarized in *Z*-direction, TTG has strong optical absorption at the range of photon energy about 3.10–4.00 eV. This result reveals that TTG have outstanding ability on harvesting visible and ultraviolet light whether along the polarization in in-plane or out-plane direction, which is superior to most of other monolayer materials.

Although the situation of its band edge positions and optical absorption indicates that TTG would be a good potential candidate for water splitting, its carrier mobility also should be concerned, which has important impact on photogenerated carrier transferring to the surface of photocatalysts and precipitating the redox reactions of water. Additionally, the obvious difference between the mobility of electrons and holes would be favourable for enhancing photocatalytic activity through improving the separation rate of photo-induced electron and hole. Thus, the carrier mobility of TTG in *X*-direction has been carried out through the deformation potential (DP) theory^41^ at 300 K:3
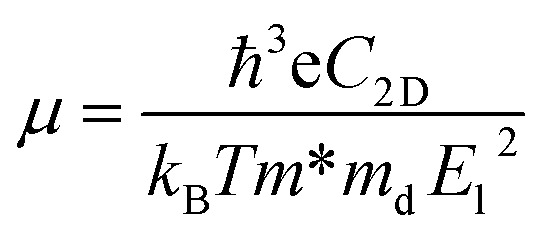
where m* represents effective mass along *X*-direction, and the *m*_d_ stands for average effective mass, which is defined as 
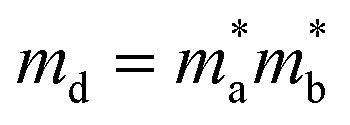
. The effective elastic modulus *C*_2D_ is calculated by *C*_2D_ = 2[∂^2^*E*/∂(Δ*a*/*a*_0_)^2^]/*S*_0_, in which the *a*_0_ is the initial lattice constant, the Δ*a* stands for the deformation of *a*_0_, the *S*_0_ represents the initial total area, and the *E* is the total energy of systems with external strain along *X*-direction. The *T* is set as 300 K. The deformation potential constant is calculated by *E*_l_ = ∂*E*_edge_/∂(Δ*a*/*a*_0_), where *E*_edge_ is the change of the band edge levels under the external strain along *X*-direction. The relevant evaluation parameters are listed in Table 1. We get that the electron mobility of TTG is ∼1208.32 cm^2^ V^−1^ s^−1^, which is higher that of monolayer MoS_2_ and GeTe. And interestingly, its hole mobility is only ∼18.69 cm^2^ V^−1^ s^−1^ mainly due to the high value of *E*_l_ for holes. The situation of carrier mobility reveals that TTG possesses favorable transport conditions of photo-induced carriers for water splitting.

**Table 1:** The relevant evaluation values for calculating carrier mobilities by deformation potential theory for TTG in *X*-direction at 300 K

TTG	m* (*m*_0_)	*M*_d_ (*m*_0_)	*E*_l_ (eV)	*C*_2D_ (eV Å^−2^)	*μ* (cm^2^ V^−1^ s^−1^)
h	1.82	1.82	7.81	11.02	18.69
e	1.77	1.77	1.00	11.02	1208.32

For photocatalytic water splitting, the solar-to-hydrogen (STH) efficiency is important target to measure the efficiency of generating hydrogen. In the case of TTG, its optical gap basically agrees with the bandgap. Therefore, the outstanding ability of harvesting light for TTG will facilitate the high STH efficiency. Firstly, we obtain the optical absorption efficiency (*η*_abs_) as follow:^42^4
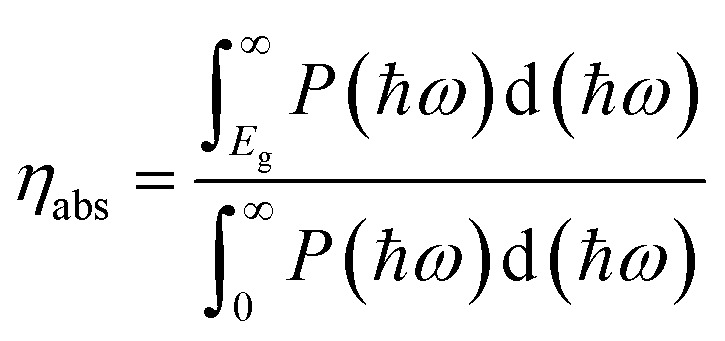
where the *P*(*ħω*) represents the AM1.5G solar energy flux that changes with the photon energy *ħω*, and the *E*_g_ stands for the bandgaps of TTG. Then we obtain that the *η*_abs_ of TTG is 31.30%. This section mainly depends on the bandgap of materials. Secondly, the carrier utilization efficiency (*η*_cu_) is obtained by following formula:^43^5
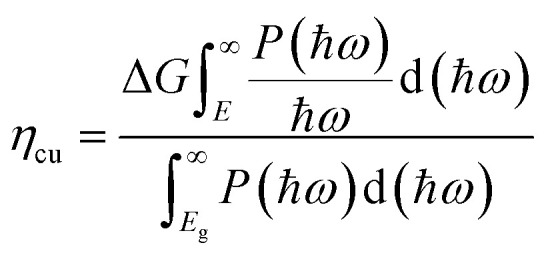
where Δ*G* = 1.23 eV is adopted, and the minimum energy *E* depending on the circumstances can be got as follow:6
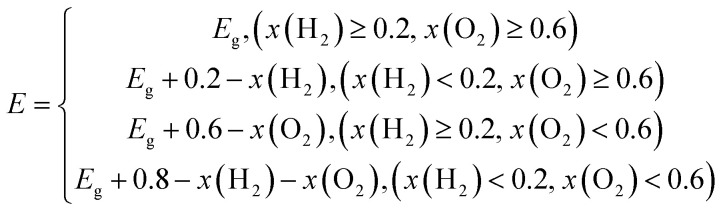
where the *x*(O_2_) stands for the driving energy of oxygen evolution reactions, and the *x*(H_2_) represents the driving energy of hydrogen evolution reactions, as shown in Fig. 3(b). For pH = 0, the *x*(O_2_), *x*(H_2_) and *E* of TTG are 0.67, 0.80 and 2.70 eV, respectively. And for pH = 7, the *x*(O_2_), *x*(H_2_) and *E* of TTG are 1.08, 0.39 and 2.70 eV, respectively. Then we carried out that the *η*_cu_ for pH = 0 and pH = 7 are 39.43% and 25.91%, respectively. Thirdly, the STH efficiency (*η*_sth_) is calculated by following expression:^44^

7 *η*_sth_ = *η*_abs_ × *η*_cu_

We obtain that the *η*_sth_ of TTG at pH = 0 is 12.34% (>10%). And although the *η*_sth_ at *v* = 7 is 8.11%, it also can be tolerable. Our calculations reveals that TTG has desirable STH efficiency for overall water splitting.

### 3.4. Strain response of TTG on electronic and photocatalytic properties

**Fig. 4:**
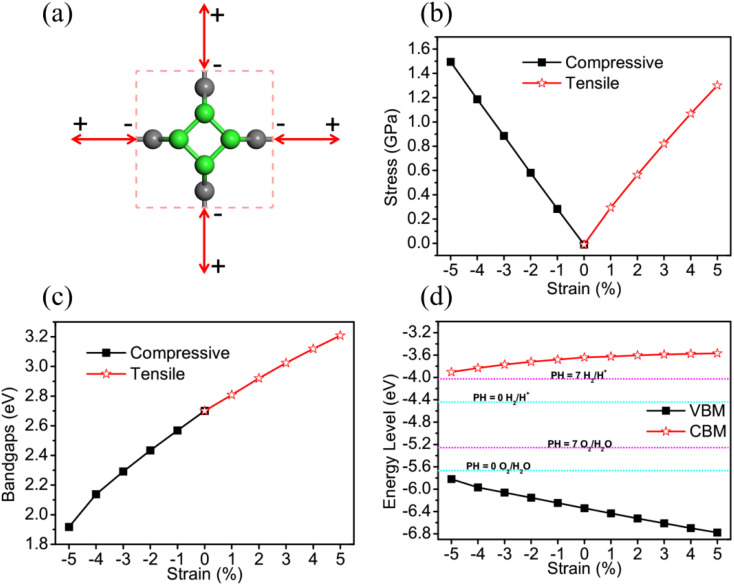
(a) The illustration of TTG with biaxial strain, in which the negative and positive values represent biaxial compressive and tensile strain, respectively. (b) and (c) are the stress and bandgaps change of TTG, respectively. (d) The VBM and CBM change of TTG compared with the standard redox vpotentials for water splitting at pH = 0 and pH = 7.

Due to predecessor's researches having proved that applying strain to 2D materials is a effective way to tune the electronic and optical properties, we have applied biaxial strain to TTG for further improving its photocatalytic properties, vas shown in Fig. 4(a). The application of biaxial strain that ranges from −5% to 5% is simulated by simultaneously freezing both lattice constant *a* and *b* at same specific values and fully relaxing carbon atoms. The biaxial strain can be defined as *η* = (*a* − *a*_0_)/*a*_0_, where *a*_0_ is the initial lattice constants, and *a* is the lattice constants with artificial strain. The Fig. 4(b) exhibits that both the tensile and compressive biaxial strain applied to TTG are experimentally feasible caused by their relatively low demand of applied stress (<1.60 GPa).

To investigate the strain response of the electronic properties of TTG, we have obtained its bandgaps and band edge positions relative to vacuum level changing with *η*. We find that, with −5% ≤ *η* ≤ 5%, the bandgap of TTG can be tuned from 1.92 eV (*η* = −5%) to 3.21 eV (*η* = 5%) and increases at a near-liner trend with the lattice constants becoming larger, as shown in Fig. 4(c). We have compared the VBM and CBM, tuned by *η*, with the redox potential of water splitting at pH = 0 and pH = 7, as shown in Fig. 4(d). The result shows that, under the biaxial strain ranging from −5% to 5%, TTG still meets the conditions of photocatalysts for water splitting at pH = 0 and pH = 7. What's more, we obtain that the energy level of VBM increases with the decrease of *η*, while it is diametrical for CBM. This phenomenon means that, with biaxial compressive strain, the optical absorption spectrum of TTG will red shift but the driving force for the redox reaction of water splitting will abate. And for the case of that with biaxial tensile strain, the relevant situation will be opposite. Furthermore, the Fig. 4(d) also exhibits that the variation of CBM with *η* is gently while it is sharp for that of VBM.

**Fig. 5:**
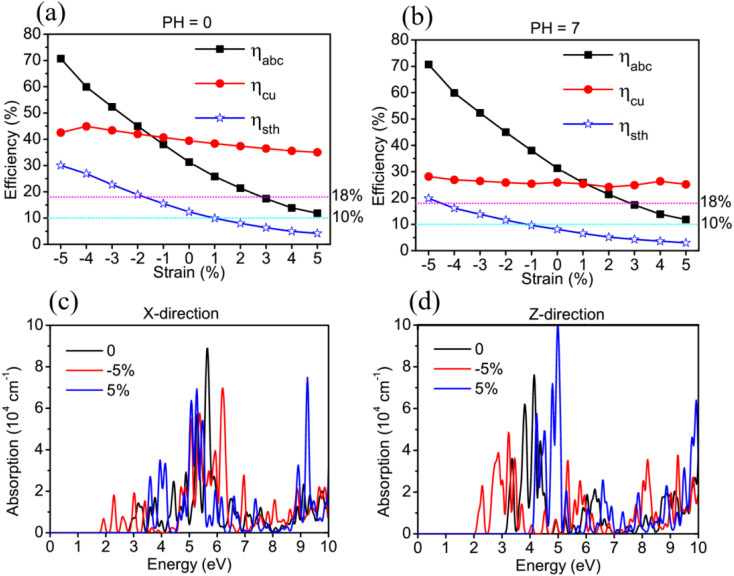
The *η*_abs_, *η*_cu_ and *η*_sth_ of TTG changing with the biaxial strain at pH = 0 (a) and pH = 7 (b). The absorption spectrum of TTG, polarized in *X*-direction (c) and *Z*-direction (d), changing with biaxial strain.

For further studying the strain effects on the photocatalytic properties of TTG, we have performed the calculations of its STH efficiency and optical absorption changing with biaxial strain. The Fig. 5(a) and (b) are the *η*_abc_, *η*_cu_ and *η*_sth_ of TTG changing with biaxial strain at pH = 0 and pH = 7, respectively. It is clear that the values of the *η*_abc_, *η*_cu_ and *η*_sth_ of TTG at pH = 0 and pH = 7 increase with the decrease of lattice constants. Moreover, as shown in Fig. 5(a), when 0 ≤ *η* ≤ 5%, the *η*_sth_ of TTG are larger than 10% at pH = 0, and when −2% ≤ *η* ≤ 5%, the *η*_sth_ of TTG are larger than 18% at pH = 0, which is the conventional theoretical limit ∼18%. Additionally, learning from Fig. 5(a), when −1% ≤ *η* ≤ 5%, the *η*_sth_ of TTG are larger than 10% at pH = 7, and when *η* = 5%, the *η*_sth_ of TTG (∼30%) is larger than 18% at pH = 7. This indicates that biaxial compressive strain is effective way improve the STH efficiency of TTG.

We investigate the strain effect on optical properties for TTG by calculating its optical absorption spectrum under biaxial strain *η* = −5%, 0 and 5%, which can present the variation trend of its optical absorption with −5%≤ *η* ≤ 5% well. The Fig. 5(c) and (d) are the optical absorption spectrums of TTG with incident light polarized in *X*-direction and *Z*-direction, respectively. We get that the optical absorption spectrum of TTG under biaxial compressive strain will be red-shift, while the situation is opposite under biaxial tensile strain. And with *η* = −5%, due to bandgap becoming smaller, the absorption spectrum of TTG is expanding to the ∼1.92 eV and have strong absorption at the range from 1.92 eV to 3.10 eV, which is conducive to solar energy conversion and visible-light photocatalytic water splitting. For the Fig. 5(d), the optical absorption spectrums of TTG with incident light polarized in *Z*-direction share same strain response feature with that with incident light polarized in *X*-direction. Furthermore, we obtain that, with *η* = −5%, the optical absorption spectrum of TTG with incident light polarized in *Z*-direction still has strong absorption at the range from 2.00 eV to 3.10 eV even surpass that with incident light polarized in *X*-direction.

### 3.5. Potential on the anode of magnesium battery

**Fig. 6:**
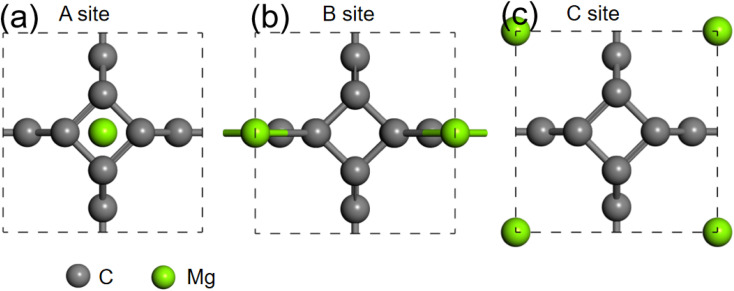
(a) is the structure of one Mg atom absorbed at above carbon tetracycline in monolayer TTG. (b) is the structure of one Mg atom absorbed onto horizontal carbon dimer in monolayer TTG. (c) is the structure of one Mg atom absorbed at the center of octagonal hole in monolayer TTG.

We further studied the use of monolayer TTG as a potential anode material for magnesium battery. The absorption energies of Mg atom on three different positions of monolayer TTG were calculated and presented in Fig. 6. The absorption energy is calculated by the following equation*E*_ab_ = *E*_C+Mg_ − *E*_C_ − *E*_Mg_where the *E*_C+Mg_ is the energy of TTG absorbing a Mg atom, the *E*_C_ is the energy of TTG, the *E*_Mg_ is the energy of a Mg atom in the bulk Mg phase. The absorption energy is 1.76 eV for A site, 0.92 eV for B site and 2.26 eV for C site. The positive adsorption energies indicate that the adsorption of Mg atom on the monolayer TTG is thermodynamically unfavorable. Hence, Mg atoms prefer being clustered rather than individually adsorbs on the monolayer TTG, similar to that of graphene–Mg complexes.^45^

**Fig. 7:**
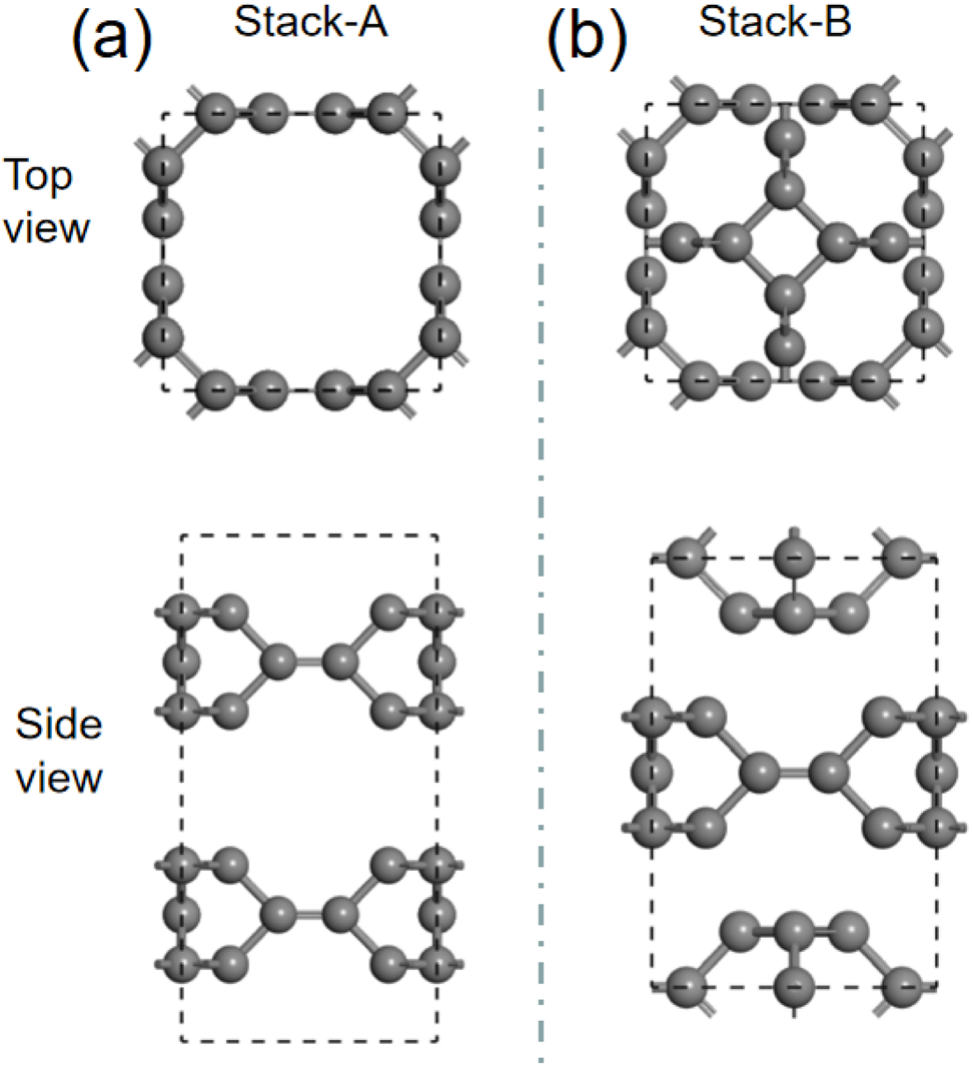
(a) is the schematic drawing of bulk TTG stacked through AA model. (b) is the system diagram of bulk TTG stacked by the way that the carbon tetracycline of one sublayer is located above the octagonal hole of the adjacent sublayers.

**Fig. 8:**
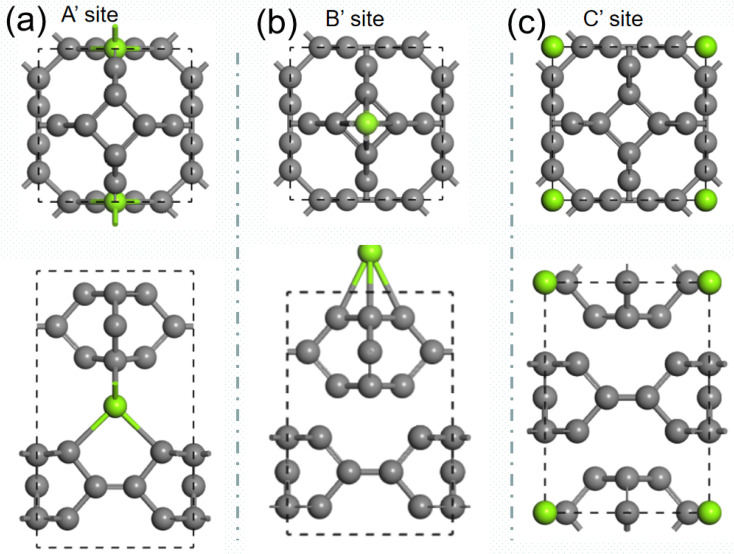
(a) is the structure of one Mg atom absorbed onto horizontal carbon dimer in bulk TTG. (b) is the structure of one Mg atom absorbed above carbon tetracycline in bulk TTG. (c) is the structure of one Mg atom absorbed at the center of octagonal hole in bulk TTG.

We then investigate the intercalation of Mg atoms into the bulk TTG. There are two stack styles of bulk TTG, as shown in Fig. 7. To identify which bulk structure is more stable, we have calculated their formation energy, and we get that the formation energy of stack-B (−0.86 eV) is much lower than that of stack-A (−0.35 eV). Therefore, the bulk TTG with stack-B structure is adopt to study the intercalation of Mg atoms. As shown in Fig. 8, three different absorption sites in bulk TTG were examined. The absorption energy is −1.15 eV for the A site, 0.41 eV for B site and 0.36 eV for C site. The negative adsorption energy of Mg atom on the A site means that intercalation of a Mg atom into the bulk TTG is thermodynamic favorable. Then, the number of Mg atoms intercalation into bulk TTG was gradually increased and the calculated adsorption energies were −1.71 eV for absorbing two Mg atoms, −1.92 eV for absorbing two Mg atoms and −1.67 for absorbing two Mg atoms, respectively. Such adsorption energies are strong enough to maintain the stability of the Mg intercalation configure without cluster. The above calculation indicates that bulk TTG can at most store Mg atoms with a stoichiometry of MgC_6_, corresponding to a theoretical capacity of 556 mA h g^−1^, which is higher than that of C_3_N and g-C_3_N_4_ sheet.^46^

To further characterizing the performance of bulk TTG as anode material of magnesium battery, we have firstly carried out the average voltage calculation using the following equation
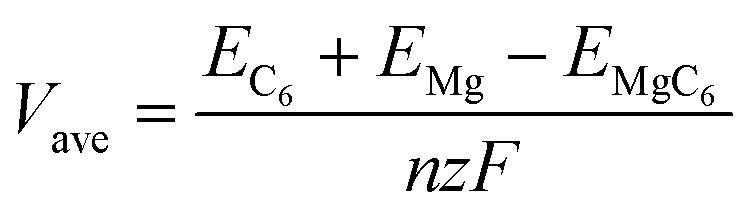
where *E*_C_6__ is the energy of the bulk TTG, *E*_Mg_ is the energy of Mg atom in the bulk Mg phase, *E*_MgC_6__ is the energy of bulk TTG with all A sites absorbing Mg atoms (named MgC_6_), *n* is the number of Mg atoms, *z* is the valence of Mg ions (+2), and *F* is the Faraday constant. The average voltage of MgC_6_ is 0.74 V, which is suitable for the application as anode of magnesium battery.

**Fig. 9:**
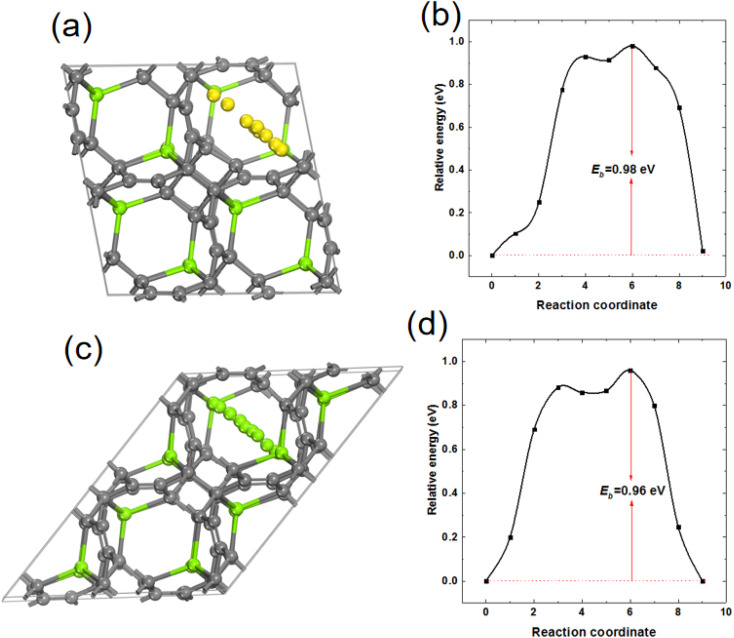
(a) and (b) are the schematic drawing of Mg atom diffusion path on the surface of TTG sublayer and corresponding Mg diffusion barrier, respectively. (c) and (d) are the schematic drawing of Mg atom diffusion path between nearby TTG sublayer and corresponding Mg diffusion barrier, respectively.

In order to study the mobility of Mg in MgC_6_, 2 × 2 × 2 supercell of MgC_6_ was used and a Mg vacancy was created from supercell MgC_6_. The energy barrier for the diffusion of a nearby Mg atom towards the Mg vacancy was calculated. As shown in Fig. 9(a) and (b), the energy barrier of Mg atom diffuses on the surface of TTG sublayer is 0.98 eV. Interestingly, as shown in Fig. 9(c) and (d), the diffusion energy barrier of Mg between nearby TTG sublayer is 0.96 eV, comparable to that of Mg on the surface of TTG sublayer. Previous computational screening of layered materials for Mg ion battery indicates the diffusion energy barrier of Mg in the layer boron carbide is 2.36 eV.^47^ The bulk TTG shows significantly low Mg diffusion energy barrier compare to that of layer boron carbide. We acknowledge that the Mg diffusion energy barrier is still too high for fast Mg ion mobility. However, our study provides a preliminary view of designed novel graphite allotropes layer materials for Mg ion battery.

## 4. Conclusion

In summary, we have studied all-sp^2^-hybridized 2D carbon allotrope TTG *via* first-principles calculations. Our calculations indicate that TTG is highly stable in energy, dynamic and thermodynamic. Through the calculations of electronic properties, we get that TTG has a wide bandgap (2.70 eV) and meets the criteria of photocatalysts for overall water splitting. The semiconductivity of TTG indicates that making π electrons localized is a key point for building all-sp^2^-hybridized 2D carbon allotrope semiconductors with considerable bandgaps. Moreover, the excellent photocatalytic behaviors, including outstanding ability of harvesting light, unique carrier mobility and desirable STH efficiency, reveal that TTG is a potential candidate for visible-light photocatalysis overall water splitting. By studying the biaxial strain response of TTG, we find that biaxial strain can effectively tune its electronic, optical and photocatalytic properties. What's more, it also reveals that biaxial compressive strain is good for improving the photocatalytic activity of TTG for overall water splitting. Finally, we have predicted that bulk TTG has potential on the application of magnesium battery as anode reflecting on suitable absorption energy, relatively lower average voltage and diffusion energy barrier. Our work provides that TTG has great potential on energy field.

## Conflicts of interest

There are no conflicts to declare.

## Supplementary Material
